# Bis-Schiff bases of 2,2′-dibromobenzidine as efficient corrosion inhibitors for mild steel in acidic medium†

**DOI:** 10.1039/c9ra06443e

**Published:** 2020-01-28

**Authors:** Ifzan Arshad, Aamer Saeed, Pervaiz Ali Channar, Syeda Aaliya Shehzadi, Muhammad Naeem Ahmed, Muhammad Siddiq

**Affiliations:** Department of Chemistry, Quaid-I-Azam University Islamabad 45320 Pakistan mifzan@gmail.com aamersaeed@yahoo.com; CAS Key Laboratory of Materials for Energy Conversion, Department of Materials Science and Engineering, University of Science and Technology of China (USTC) JinZhai Road Hefei Anhui Province 230026 P. R. China; Sulaiman Bin Abdullah Aba Al-Khail-Centre for Interdisciplinary Research in Basic Sciences (SA-CIRBS), International Islamic University Islamabad Pakistan; Department of Chemistry, The University of Azad Jammu and Kashmir Muzaffarabad 13100 Pakistan

## Abstract

In this work, three new bis-Schiff bases, namely 1,1′-(2,2′-dibromo-[1,1′-biphenyl]-4,4′-diyl)bis(*N*-phenylmethanimine) (BNSB01), 1,1′-(2,2′-dibromo-[1,1′-biphenyl]-4,4′-diyl)bis(*N*-(4-bromophenyl)methanimine) (BNSB02) and 4,4′-(((2,2′-dibromo-[1,1′-biphenyl]-4,4′-diyl)bis(methanylylidene))bis(azanylylidene))diphenol (BNSB03), were synthesized. These Schiff bases were evaluated for their corrosion inhibition ability on mild steel specimens in 0.5 M HCl by using electrochemical and weight loss techniques. The inhibition performance was found to increase with an increase in the inhibitor concentration and decrease with an increase in temperature. The results revealed that the synthesized compounds followed the Langmuir isotherm model and were efficient mixed-type inhibitors. The electrochemical impedance studies also indicated that with a rise in the concentration of inhibitors, the charge transfer resistance increased. The surface morphology of the inhibited and uninhibited specimens was examined using scanning electron microscopy (SEM). The efficiency of the compounds was in the order BNSB02 > BNSB03 > BNSB01. All the results obtained were in good correlation with each other.

## Abstract 2

The inhibitory effect of three new bis-Schiff bases on mild steel corrosion in 0.5 M HCl was studied by the weight loss method and the electrochemical method.

## Graphical abstract

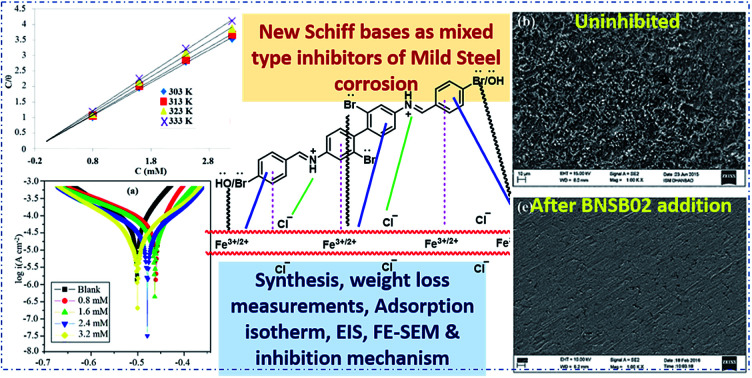

## 1. Introduction

Mild steel is one of the versatile, extensively used and least-expensive materials used in nearly all kinds of industries. One of the principal issues in an industrial process is the inevitable damage of metals and steel due to corrosion, which prompts an upsurge in the manufacturing cost. There are numerous techniques for the control and prevention of corrosion in rough environments.^1–3^ Nowadays, a cost-effective method employing organic inhibitors is applied to decrease corrosion attack.^4^ Generally, these corrosion inhibitors are used in small quantities. Many investigations^5–8^ have proven that nitrogen-, phosphorous-, oxygen-, and sulfur-containing organic compounds are the most effective for this purpose. Their corrosion inhibition activity is generally accredited to their interactions with the metal surface. Usually, the active corrosion sites are blocked by the adsorption of organic compounds on the surface of the metal.

Among the various hetero-atom containing compounds, Schiff bases are quite effective because of the presence of a nitrogen atom, and several such compounds have been reported in the literature as potential corrosion inhibitors for metals and alloys in an acidic medium.^9–13^ The growing popularity of Schiff bases as corrosion inhibitors is primarily based on their low toxicity and convenience of synthesis from very inexpensive starting materials.^14,15^ Schiff bases are^1,16–21^ well-known for their potential for corrosion inhibition, and plentiful research on organic inhibitors has shown that Schiff bases have much greater inhibition efficiencies compared to the analogous aldehydes and amines.

The above considerations encouraged us to evaluate the thermodynamic parameters of the adsorption of three synthesized Schiff bases, namely 1,1′-(2,2′-dibromo-[1,1′-biphenyl]-4,4′-diyl)bis(*N*-phenylmethanimine) (BNSB01), 1,1′-(2,2′-dibromo-[1,1′-biphenyl]-4,4′-diyl)bis(*N*-(4-bromophenyl)methanimine) (BNSB02) and 4,4′-(((2,2′-dibromo-[1,1′-biphenyl]-4,4′-diyl)bis(methanylylidene))bis(azanylylidene))diphenol (BNSB03), on the mild steel surface in 0.5 M HCl using weight loss measurements and electrochemical techniques.

## 2. Experimental methods

### 2.1 Materials and sample preparation

Analytical grade chemicals and solvents were used for the synthesis of the bis-Schiff bases. All the solvents, including benzaldehyde, 4-bromobenzaldehyde, and 4-hydroxybenzaldehyde, were purchased from Sigma Aldrich and used without further purification. GR grade HCl (35%) was obtained from Merck. Mild steel specimens with an elemental composition of C: 0.17%, Si: 0.59%, Mn: 1.6%, P: 0.040% and iron for the rest were used. Specimens of dimensions 2 × 2 × 0.1 cm were used for all experiments. Before the commencement of electrochemical and gravimetric experiments, the mild steel specimens were polished with emery paper of 600, 800 and 1200 grades under running tap water. These were washed with distilled water, dried with a clean tissue, and finally immersed in benzene and acetone for a few seconds before air drying.^22,23^

### 2.2 Synthesis and characterization data of the corrosion inhibitors

**Fig. 1:**
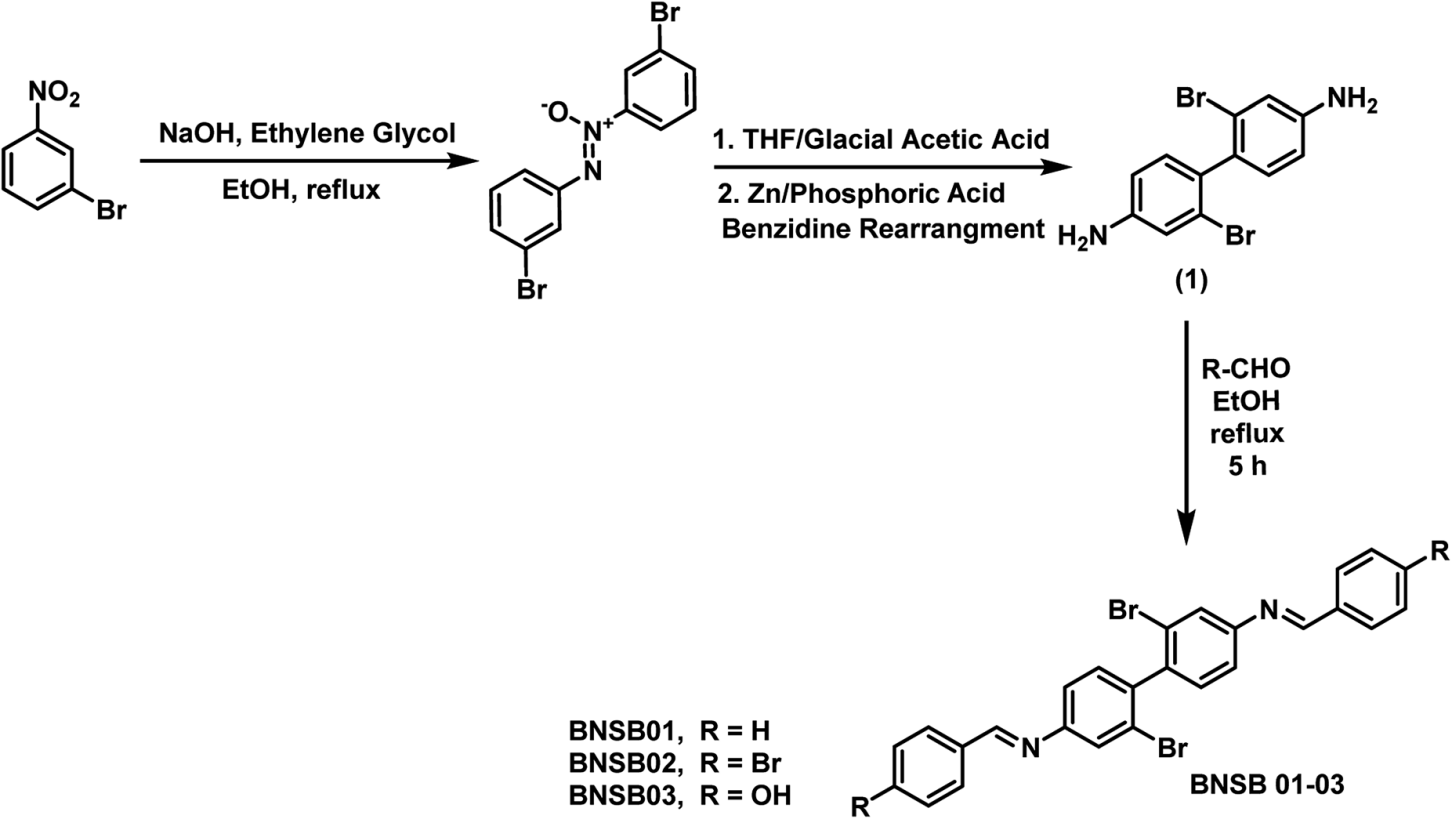
Synthetic route of the Schiff bases BNSB01, BNSB02 and BNSB03.

The compound 2,2′-dibromo-4,4′-benzidine (1), as shown in Fig. 1, was synthesized according to a procedure published earlier.^24^ The Schiff bases were synthesized by refluxing two moles of benzaldehyde, namely 4-bromobenzaldehyde and 4-hydroxybenzaldehyde, separately with one mole of 2,2′-dibromo-4,4′-benzidine (1) each using ethanol as the solvent for 5 h. The mixture was cooled to room temperature and filtered to obtain the resultant solid, which was further vacuum-dried. The synthetic scheme and molecular structures of the synthesized Schiff bases (BNSB01, BNSB02 and BNSB03) are shown in Fig. 1.

#### 2.2.1 1,1′-(2,2′-Dibromo-[1,1′-biphenyl]-4,4′-diyl)bis(*N*-phenylmethanimine) (BNSB01)

Yield: 93%, elemental analysis: calc.: C, 60.26; H, 3.50; Br, 30.84; N, 5.41; found: C, 60.19; H, 3.42; Br, 30.77; N, 5.33%; characteristic IR peaks (KBr disk): *ν*C–H (aromatic) = 3046, *ν*C=N = 1624, *ν*C=C = 1496, *ν*C–Br = 570 cm^−1^; ^1^H NMR (400 MHz, CDCl_3_, *δ*, ppm) 8.52 (s, 2H, N=C–H), 7.93–7.92 (m, 4H, Ar–H), 7.53–7.51 (m, 8H, Ar–H), 7.31–7.23 (m, 4H, Ar–H); ^13^C NMR (100 MHz, CDCl_3_, *δ*, ppm) *δ* = 159.57, 143.72, 142.47, 136.01, 128.93, 128.88, 128.75, 127.83, 123.53, 120.74, 117.85.

#### 2.2.2 1,1′-(2,2′-Dibromo-[1,1′-biphenyl]-4,4′-diyl)bis(*N*-(4-bromophenyl)methanimine) (BNSB02)

Yield: 88%, elemental analysis: calc.: C, 46.19; H, 2.39; Br, 47.28; N, 4.14; found: C, 46.11; H, 2.34; Br, 46.02; N, 4.07%; characteristic IR peaks (KBr disk): *ν*C–H (aromatic) = 3049, *ν*C=N = 1623, *ν*C=C = 1491, *ν*C–Br = 571 cm^−1^; ^1^H NMR (400 MHz, CDCl_3_, *δ*, ppm) 8.46 (s, 2H, N=C–H), 7.80–7.78 (d, 4H, Ar–H), 7.64, 7.62 (d, 4H, Ar–H), 7.53–7.52 (d, 2H, Ar–H), 7.30–7.22 (m, 4H, Ar–H); ^13^C NMR (100 MHz, CDCl_3_, *δ*, ppm) 159.59, 143.74, 142.49, 135.02, 131.93, 130.13, 127.85, 124.05, 123.55, 120.76, 117.87.

#### 2.2.3 4,4′-(((2,2′-Dibromo-[1,1′-biphenyl]-4,4′-diyl)bis(methanylylidene))bis(azanylylidene))diphenol (BNSB03)

Yield: 91%, elemental analysis: calc.: C, 56.75; H, 3.30; Br, 29.04; N, 5.09; O, 5.82 found: C, 56.69; H, 3.23; Br, 28.98; N, 5.00; O, 5.75%; characteristic IR peaks (KBr disk): *ν*C–H (aromatic) = 3045, *ν*C=N = 1625, *ν*C=C = 1493, *ν*C–Br = 572, *ν*O–H = 3450 cm^−1^; ^1^H NMR (400 MHz, EtOD, *δ*, ppm) *δ* = 8.37 (s, 2H, N=C–H), 7.77–7.73 (m, 4H, Ar–H), 7.48–7.46 (d, 2H, Ar–H), 7.23–7.16 (m, 4H, Ar–H), 6.92–6.89 (m, 4H, Ar–H); ^13^C NMR (100 MHz, EtOD, *δ*, ppm) *δ* = 159.57, 157.84, 143.72, 142.47, 129.28, 128.76, 127.83, 123.53, 120.74, 117.85, 115.02.

### 2.3 Weight loss measurements

The mild steel specimens were dipped in a solution of 0.5 M HCl with variable amounts of the inhibitors for 4 h in a thermostatically controlled water bath at a constant temperature under aerated conditions, and for comparison purposes, the control was also established. After a specified time interval, these specimens were taken out and rinsed gently with water and acetone until the corrosion products on the specimens were rinsed thoroughly. The average weight loss was calculated by performing a triplicate experiment. The same procedure was repeated for different combinations of temperatures and concentrations of the inhibitors.

### 2.4 Electrochemical measurements

Electrochemical impedance spectroscopy (EIS) and potentiodynamic polarization studies were performed using an electrochemical workstation CHI660D. For this purpose, a conventional cell consisting of three-electrodes, namely a reference electrode (Ag/AgCl), an auxiliary platinum electrode and a working electrode of mild steel with 1 cm^2^ area, was used. The electrochemical measurements were performed using all three bis-Schiff base derivatives at 30 °C with variable inhibitor concentrations (0.8–3.2 mM). Potentiodynamic polarization measurements were carried out in the range of −850 to −150 mV at a 0.4 mV s^−1^ scan rate. Before EIS measurements, the open circuit potential was stabilized for 30 min. EIS data were recorded between 1 Hz to 100 kHz frequency.

### 2.5 Field emission scanning electron microscopy (FE-SEM)

The surface morphology of the mild steel specimens was studied by using a field emission scanning electron microscope (FE-SEM) Supra 55 (Carl Zeiss, Germany) at a 10 μm scale with 1.0k× magnification. The samples were immersed in 0.5 M HCl, and the effect of different inhibitors along with the control was studied. After 4 h of immersion under optimum conditions, SEM images of both polished mild steel specimens and specimens immersed in 0.5 M HCl in the presence and absence of inhibitors were captured.

## 3. Results and discussion

To synthesize the corrosion inhibitors BNSB01, BNSB02, and BNSB03, the sequence of reactions outlined in Fig. 1 was followed. First, 1-bromo-3-nitrobenzene was converted to an azoxy compound by reduction, which underwent benzidine rearrangement in the presence of zinc dust to form 2,2′-dibromo-4,4′-benzidine (1). The desired Schiff base inhibitors were prepared by the reaction of benzidine (1) with benzaldehyde, 4-bromobenzaldehyde and 4-hydroxybenzaldehyde under reflux in ethanol. The synthesized compounds were characterized by NMR (^1^H and ^13^C) spectroscopy.

### 3.1 Weight loss measurements

#### 3.1.1 Effect of inhibitor concentration

The rate of corrosion (*C*_R_) and percentage inhibition efficiency (IE%) of the inhibitors BNSB01, BNSB02, and BNSB03 at different concentrations (0.8–3.2 mM) and temperatures (30 to 60 °C) were determined by weight loss measurements, and the data are illustrated in Table 1. The rates of corrosion and inhibition efficiency (%) were determined by using the following eqn (1) and (2).1
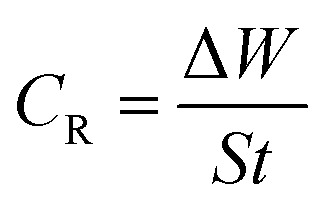
where *S* is the total exposed area (cm^2^), Δ*W* is the weight loss, and *t* is the time of exposure (h) of the specimen.2
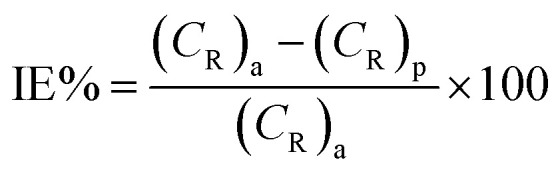
where (*C*_R_)_a_, and (*C*_R_)_p_ are the rates of corrosion in the absence and presence of inhibitors, respectively.

**Table 1:** Weight loss data of mild steel after 4 h immersion in 0.5 M HCl in the absence and presence of different concentrations (*C*) of the inhibitors at different temperatures

Inhibitor	*C* (mM)	30 °C *C*_R_ (mg cm^2^ h^−1^)	IE (%)	40 °C *C*_R_ (mg cm^−2^ h^−1^)	IE (%)	50 °C *C*_R_ (mg cm^2^ h^−1^)	IE (%)	60 °C *C*_R_ (mg cm^−2^ h^−1^)	IE (%)
	Blank	0.516	—	0.883	—	1.224	—	1.65	—
BNSB01	0.8	0.144	72.1 ± 1.11	0.28	68.3 ± 0.78	0.449	63.3 ± 0.55	0.669	59.5 ± 0.6
	1.6	0.128	75.2 ± 0.94	0.255	71.1 ± 0.87	0.389	68.2 ± 0.18	0.575	65.1 ± 0.84
	2.4	0.100	80.6 ± 0.38	0.202	77.1 ± 0.66	0.337	72.4 ± 0.44	0.515	68.8 ± 0.77
	3.2	0.074	85.6 ± 0.55	0.163	81.5 ± 0.68	0.287	76.6 ± 0.55	0.453	72.5 ± 0.58
BNSB02	0.8	0.113	78.1 ± 0.62	0.218	75.3 ± 0.44	0.348	71.6 ± 0.38	0.534	67.6 ± 0.84
	1.6	0.097	81.2 ± 0.84	0.184	79.2 ± 0.78	0.307	74.9 ± 0.44	0.48	70.9 ± 0.52
	2.4	0.074	85.7 ± 0.43	0.143	83.8 ± 0.84	0.268	78.1 ± 0.51	0.422	74.4 ± 0.46
	3.2	0.050	90.3 ± 1.20	0.113	87.1 ± 1.40	0.211	82.7 ± 0.35	0.367	77.7 ± 0.69
BNSB03	0.8	0.128	75.1 ± 0.56	0.256	71.0 ± 1.16	0.416	66.0 ± 0.24	0.583	64.7 ± 0.86
	1.6	0.108	78.9 ± 0.28	0.237	73.1 ± 0.83	0.357	70.8 ± 1.10	0.56	66.1 ± 0.23
	2.4	0.091	82.3 ± 0.79	0.193	78.1 ± 0.74	0.331	72.9 ± 0.46	0.477	71.1 ± 1.12
	3.2	0.065	87.4 ± 0.96	0.154	82.5 ± 0.45	0.273	77.6 ± 0.68	0.413	75.0 ± 1.50

The inhibition efficiency increased on increasing the inhibitor concentration, and a decrease in the rate of corrosion was observed at all concentrations (*i.e.* 0.8–3.2 mM), as shown in Table 1. Therefore, it is evident that the inhibition efficiency was concentration dependent. With increasing inhibitor concentration, greater numbers of molecules are adsorbed on the surface of mild steel, which results in increased inhibition efficiency. The adsorbed molecules block the reaction sites and thus protect the metal from corrosion. As the Schiff bases had sufficient available electrons, *i.e.* a lone pair on nitrogen, a lone pair on bromine and the π electrons, which are strongly bonded to the positively-charged metal surface, they could inhibit corrosion. At a concentration of 3.2 mM and 30 °C, BNSB01, BNSB02 and BNSB03 showed the maximum inhibition efficiencies of 85.6%, 90.3% and 87.4%, respectively. Beyond this, no increase in inhibition efficiency was observed with a further increase in inhibitor concentration. The results demonstrated the inhibition efficiencies were in the order BNSB02 > BNSB03 > BNSB01. Visual observation before and after the experiment demonstrated that the mild steel specimen almost retained its bright exterior in the presence of inhibitors, whereas the ones immersed into the acid solution did not. This observation endorsed that these inhibitors were highly effective in suppressing corrosion attacks and the rate of corrosion.

#### 3.1.2 Effect of temperature

Temperature significantly influences the rate of corrosion, and with rising temperature, the corrosion rates increase exponentially in an acidic medium (hydrogen depolarisation) while the hydrogen evolution overpotential decreases.^25^ To comprehend the inhibition efficiency of the Schiff base inhibitors at higher temperatures, their weight loss was measured from 30–60 °C. All the three compounds showed the maximum inhibition efficiency at 30 °C, which steadily decreased with a further rise in temperature. The examined inhibitors showed lower efficiency at higher temperatures because the increase in temperature did not support physical interactions, thus lowering the inhibition efficiency. The adsorption and desorption process at higher temperatures happens after a little time gap, and the duration of exposure of the metal surface to the acidic environment is longer, which lowers the inhibition efficiency.^26^

The relationship of temperature with the rate of corrosion could be given by the Arrhenius type eqn (3):3
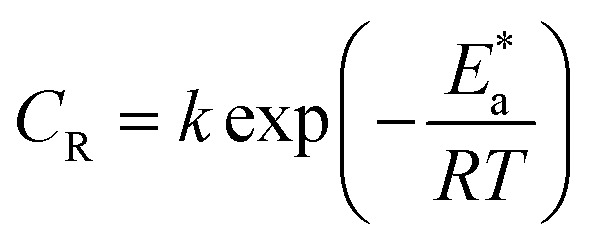


**Fig. 2:**
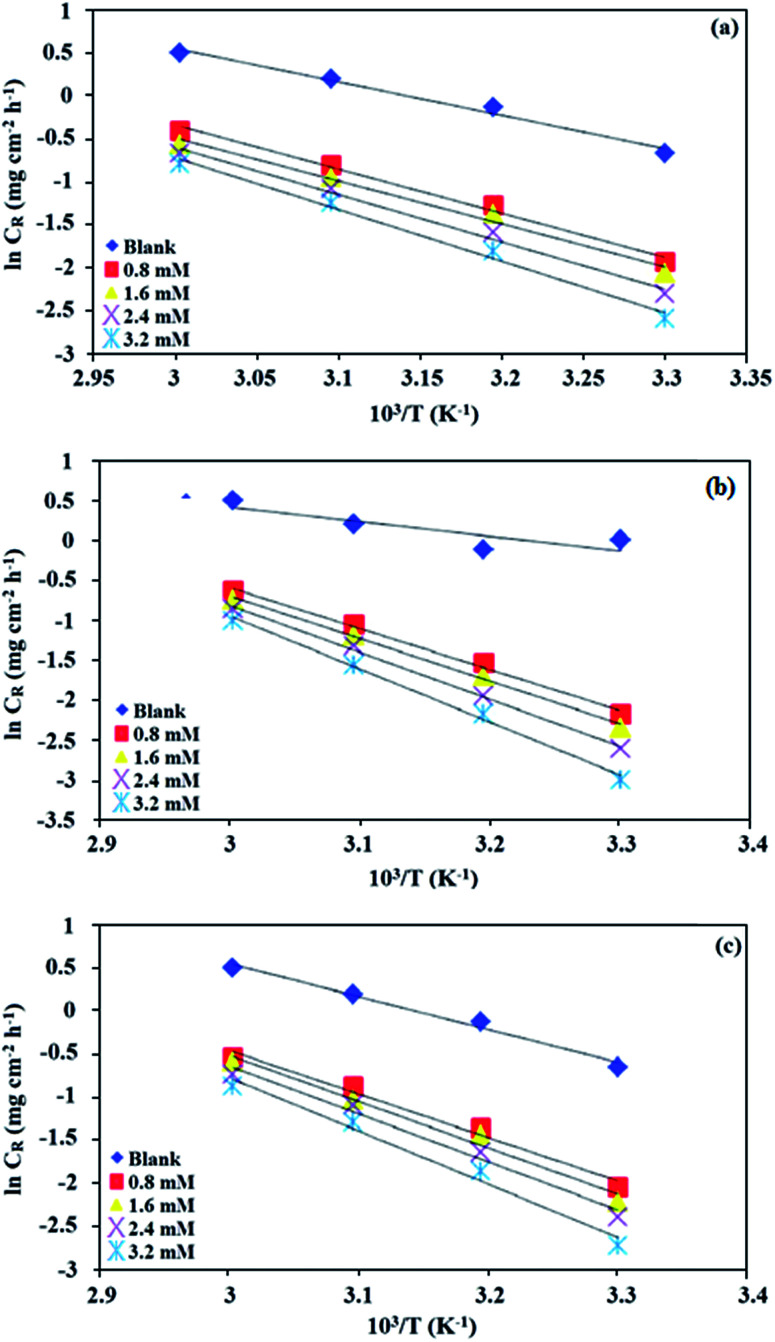
Arrhenius plots for mild steel in 0.5 M HCl in the absence and presence of different concentrations of (a) BNSB01, (b) BNSB02 and (c) BNSB03.

An alternative to the Arrhenius equation was4
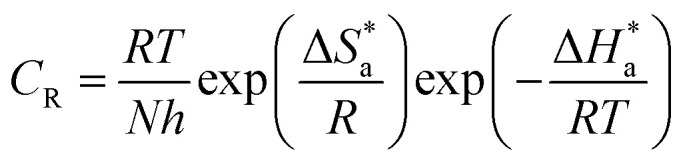
where 
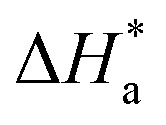
 is the enthalpy of activation, 
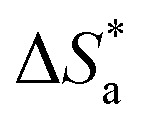
 is the entropy of activation, 
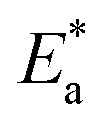
 is the energy of activation, *k* is the Arrhenius pre-exponential factor, *N* is Avogadro's number, *h* is Planck's constant, *R* is the universal gas constant, and *T* is the absolute temperature. The 
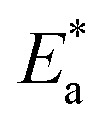
 and *k* values for mild steel at different concentrations with and without inhibitors were calculated from the values of the slope and intercept of the Arrhenius plots of log *C*_R_ against 1/*T* (Fig. 2), respectively, drawn using eqn (3). 
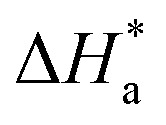
 and 
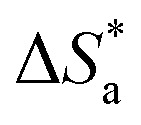
 were calculated from the slope 
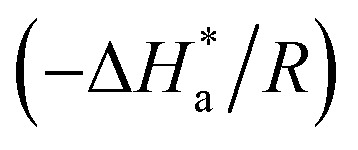
 and intercept 
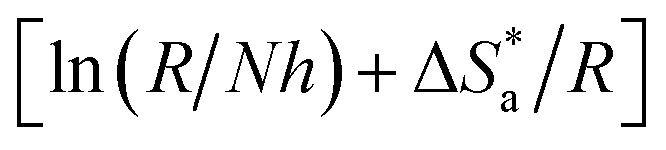
 of the ln *C*_R_/*T vs.* 1/*T* plots (Fig. S1 in ESI†) drawn using eqn (4). Table 2 presents all the values of 
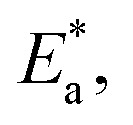
*k*, 
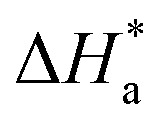
 and 
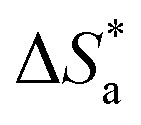
 for the blank and in the presence of inhibitors.

**Table 2:** Activation parameters of mild steel in the absence and presence of inhibitors at different temperatures

Inhibitor	*C* (mM)	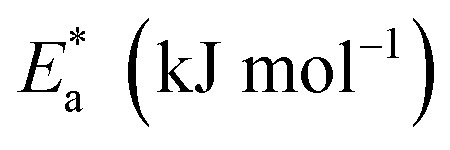	*k* (mg cm^−2^ h^−1^)	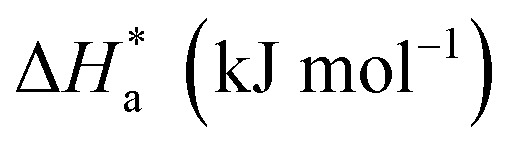	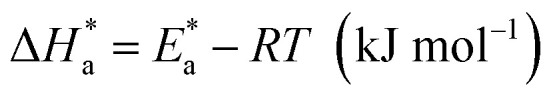	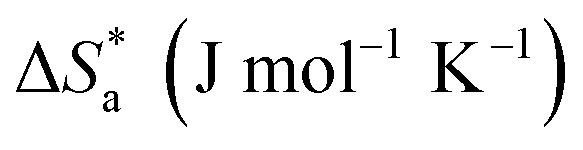
	Blank	32.10	186 465	29.45	29.49	−152.85
BNSB01	0.8	42.71	3 506 048	40.08	40.11	−128.41
	1.6	41.52	1 963 030	38.89	38.92	−133.21
	2.4	45.7	8 008 388	43.06	43.1	−121.59
	3.2	50.45	39 586 551	47.81	47.85	−108.30
BNSB02	0.8	43.15	3 259 225	40.51	40.55	−129.06
	1.6	44.68	5 050 511	42.03	42.07	−125.43
	2.4	49.23	23 277 552	46.59	46.63	−112.71
	3.2	55.53	196 662 575	52.88	52.93	−94.97
BNSB03	0.8	42.31	2 716 894	30.48	39.70	−130.58
	1.6	44.78	6 230 705	39.66	42.18	−123.68
	2.4	46.33	9 597 373	42.14	43.73	−120.09
	3.2	51.47	53 704 105	43.69	48.87	−105.77

The activation energy calculations revealed that the value of 
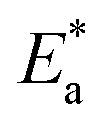
 was higher in the presence of inhibitors compared to that of the blank, which was 32.10 kJ mol^−1^. The higher values of 
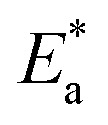
 were attributed to the formation of inhibitor-metal complexes in the acidic medium with higher energy barriers.^27^ The decrease in the inhibition efficiency with rising temperature was because the *E*_a_ of the inhibitor solution was greater than that of the blank.^28,29^ The outcomes of this study rationalize the statement that *E*_a_ increases with an increase in temperature due to the reduction in physisorption.^30^ The positive 
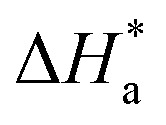
 values in the presence of inhibitors suggested the endothermic dissolution of mild steel, which is a difficult task.^30,31^ The value of 
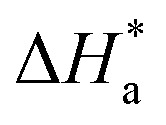
 in the absence of inhibitors was 29.45 kJ mol^−1^, which was less than the values (40.11–52.93 kJ mol^−1^) calculated in the presence of the three inhibitors.

The large negative values of 
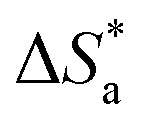
 in the presence of inhibitors indicated the associative formation of activated complexes. In total, the sum of the increased entropy caused by the desorption of solvent molecules (water) and the decrease in entropy due to the adsorption of organic molecules (solute) is equal to the entropy of activation.^32^ In the presence of inhibitors, the activation entropy changes because of the adsorption and desorption process of the inhibitors and water molecules on the surface of steel.^33^

### 3.2 Adsorption isotherms

The interaction between the corrosion inhibitors and steel were best examined by the adsorption isotherms resulting from chemisorption or physisorption. Mainly, the rate of corrosion is affected by the degree of surface coverage of the inhibitors. Therefore, the inhibition efficiency is termed as the function of the electrode surface covered by the molecules of inhibitors.^34^ The concentration and degree of surface coverage (*θ*) were used to determine the linear relation of the adsorption isotherm (*θ* = IE (%)/100). Various adsorption isotherms, such as Frumkin, Temkin, and Langmuir, were tested for a better understanding of the behavior of the inhibitors, and the Langmuir isotherm model was the best fit.

**Fig. 3:**
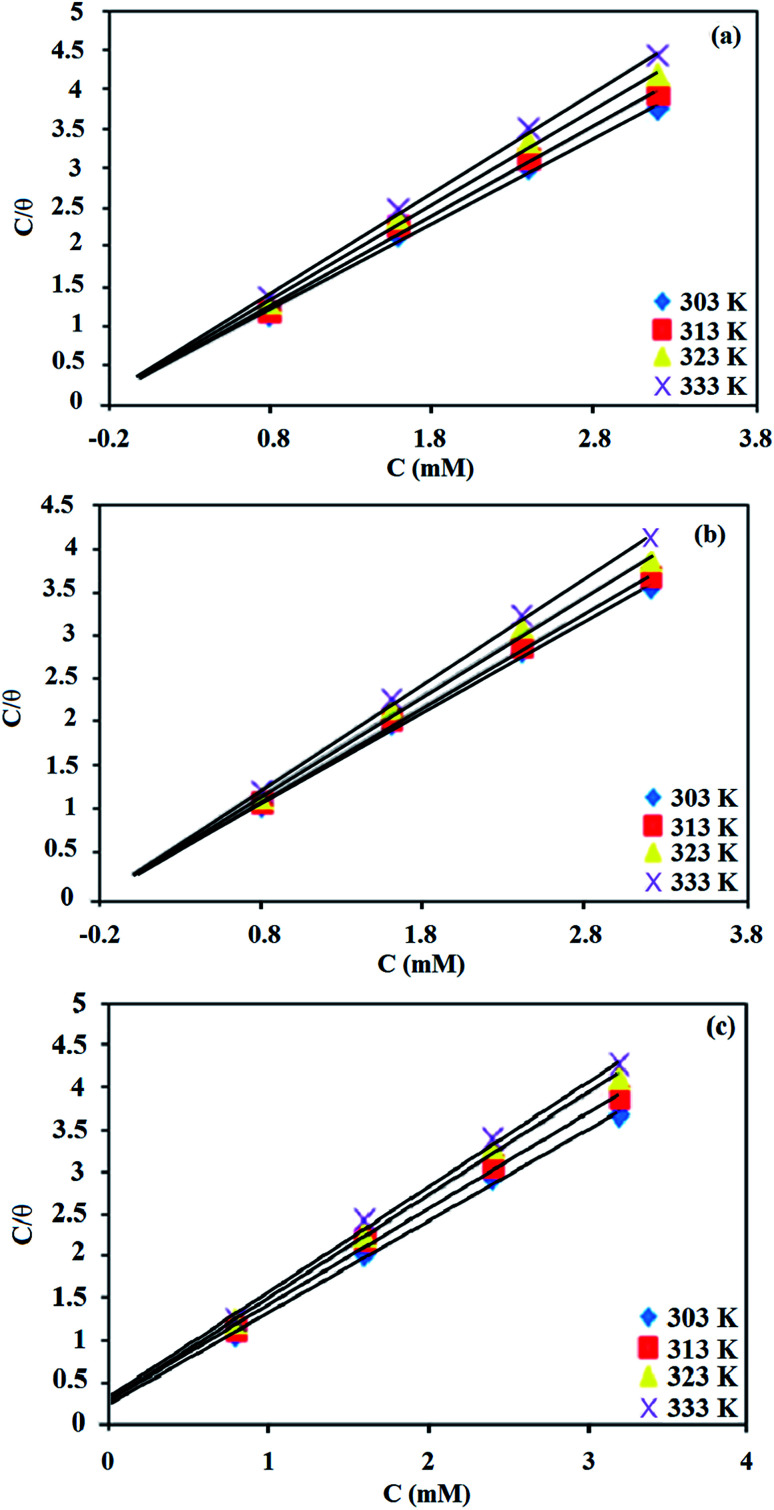
Langmuir isotherms for the adsorption of (a) BNSB01, (b) BNSB02 and (c) BNSB03 on mild steel in 0.5 M HCl at different temperatures.

The relationship between *θ* and *C* was given by eqn (5).5
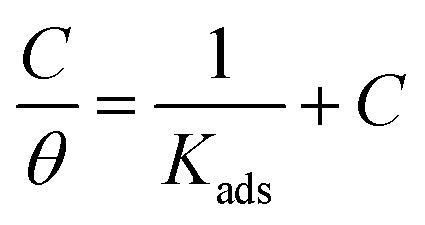
where *C* is the inhibitor concentration, *θ* is the covered surface, and *K*_ads_ is the equilibrium constant. When a graph was plotted between *C* and *C*/*θ*, a straight line with a regression coefficient of 0.99 and a slope of 1 was obtained, as shown in Fig. 3. The linear relationship proposes that the inhibitors adsorbed on the mild steel surface obeyed the Langmuir adsorption isotherm and had no interaction with the neighboring sites.^35,36^

*K*_ads_ was determined using eqn (5), and the free energy of adsorption was calculated from *K*_ads_ using eqn (6).6
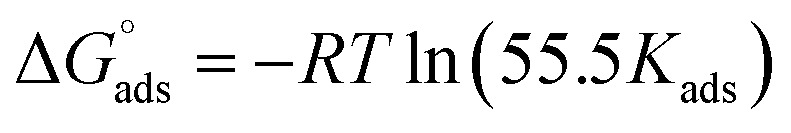
where *T* is the temperature, *R* is the universal gas constant, and the concentration of water was 55.5 mol dm^−3^. 
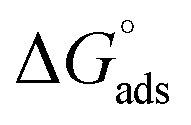
 calculated using eqn (6) was negative and ranged between −30.50 to −34.05 kJ mol^−1^. These values suggested that the adsorption of inhibitors on the surface of the metal was a spontaneous process. Previously, it has been reported that less negative values of 
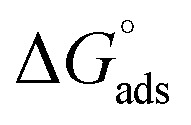
 (around −20 kJ mol^−1^) indicate adsorption mainly due to electrostatic interactions, and values of 
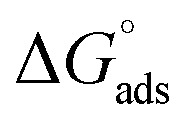
 around −40 kJ mol^−1^ suggest chemisorption. The values of 
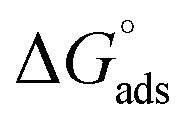
 in this study were between −20 kJ mol^−1^ and −40 kJ mol^−1^, which suggested both types of adsorptions *i.e.* physisorption and chemisorption.

The enthalpy and entropy of adsorption were calculated using eqn (7):7
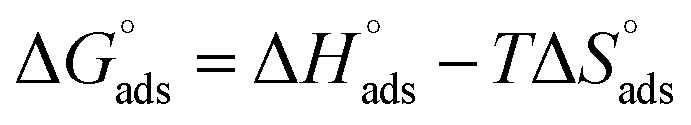


The plot of *T* against 
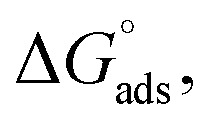
 exhibited a straight-line with intercept 
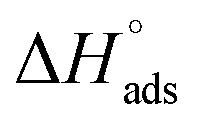
 and slope – 
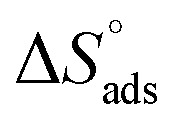
 (Fig. S2†). Table 3 shows the values of the thermodynamic parameters for the three inhibitors. Positive values for the entropy of adsorption indicated that the entropy of the solvent prevailed over the entropy of the solutes. It has also been reported that the type of adsorption can be determined from the value of 
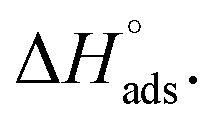
 If 
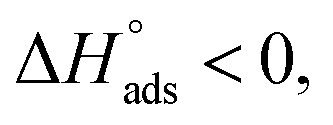
 then it could be physisorption or chemisorption, and if 
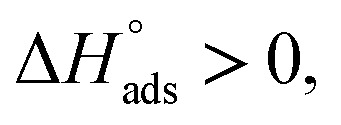
 the type of adsorption is chemisorption.^37^ Usually, an enthalpy of adsorption lower than 40 kJ mol^−1^ is associated with chemisorption and that higher than 100 kJ mol^−1^ is associated with physisorption.^38^ For all of these Schiff bases, the enthalpy of adsorption was negative *i.e.* −2.14 to −5.023 kJ mol^−1^, which endorsed the mode to be physisorption.

**Table 3:** Thermodynamic parameters for the adsorption of BNSB01, BNSB02 and BNSB03 on mild steel in 0.5 M HCl at different temperatures from the Langmuir adsorption isotherms

Inhibitor	*T* (K)	*R* ^2^	*K*_ads_ (L mol^−1^)	Δ*G*_ads_ (kJ mol^−1^)	Δ*S*_ads_ (J mol^−1^ K^−1^)	Δ*H*_ads_ (kJ mol^−1^)	Δ*G*_ads_ = Δ*H*_ads_ − *T*Δ*S*_ads_ (kJ mol^−1^)
BNSB01	303	0.996	3276.5	−30.50	84	−5.023	−30.47
	313	0.995	3003	−31.28			−31.31
	323	0.997	2879.3	−32.17			−32.15
	333	0.998	2720.3	−33.01			−32.99
BNSB02	303	0.997	4221.1	−31.14	95.8	−2.142	−31.16
	313	0.998	4233.7	−32.18			−31.12
	323	0.997	3968.2	−33.03			−33.08
	333	0.998	3961.9	−34.05			−34.05
BNSB03	303	0.997	4042	−31.03	82.8	−5.89	−30.98
	313	0.996	3544.8	−31.72			−31.81
	323	0.997	3554.9	−32.74			−32.64
	333	0.995	3193.8	−33.45			−33.46

### 3.3 Potentiodynamic polarization studies

**Fig. 4:**
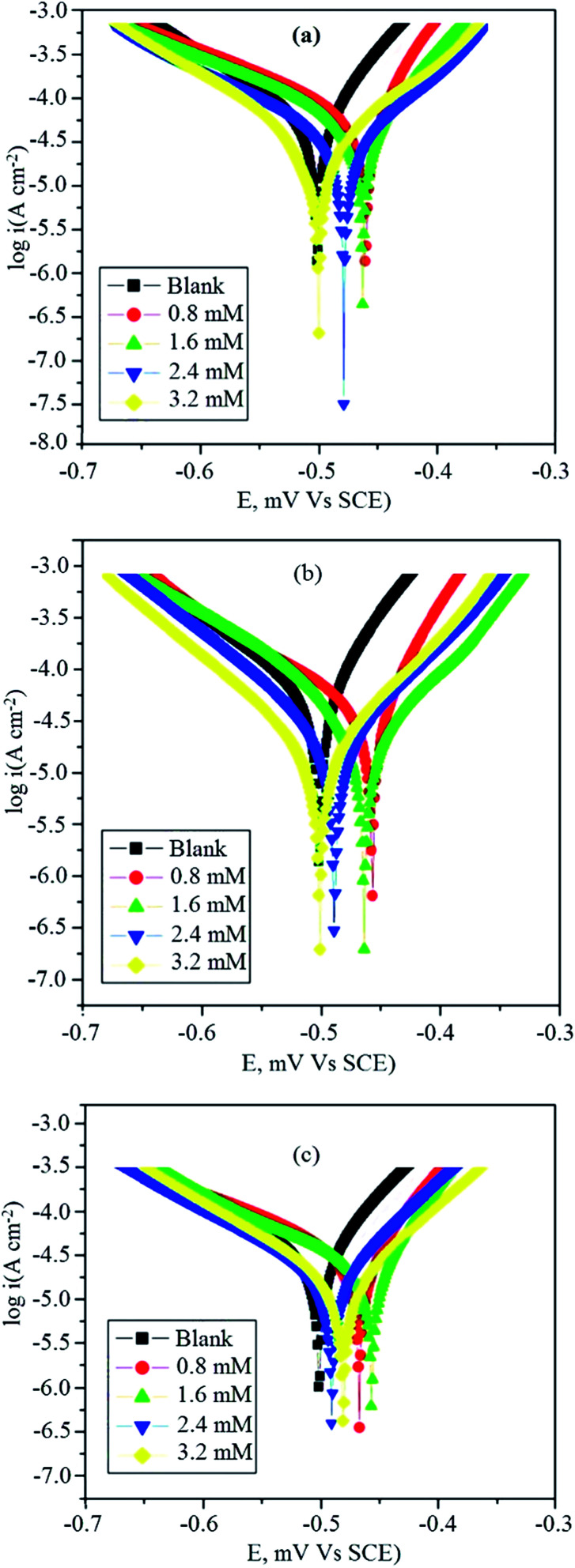
Polarisation curves of mild steel in 0.5 M HCl containing different concentrations of (a) BNSB01, (b) BNSB02 and (c) BNSB03.

Potentiodynamic polarization studies were carried out for a good understanding of the behavior of the inhibitors in their bias toward the anodic and cathodic reactions. The anodic and the cathodic Tafel curves for BNSB01, BNSB02, BNSB03 and blank are presented in Fig. 4. Table 4 shows the parameters of electrochemical corrosion kinetics, *i.e.* Tafel slopes (*b*_a_, *b*_c_), corrosion potential (*E*_corr_), corrosion current density (*i*_corr_), and linear polarization.

**Table 4:** Potentiodynamic polarisation parameters for the corrosion of mild steel in 0.5 M HCl in the absence and presence of different concentrations of BNSB01, BNSB02 and BNSB03 at 303 K

Inhibitor	Concentration (mM)	−*E*_corr_ (mV)	*i*_corr_ (μA cm^−2^)	*b*_a_ (mV dec^−1^)	−*b*_c_ (mV dec^−1^)	Linear polarisation resistance (Ω cm^2^)	IE (%)
	Blank	502	200	4.538	2.658	302	—
BNSB01	0.8	461	64.1	10.784	5.735	294.5	67.95
	1.6	463	54.5	13.258	5.644	422.2	72.77
	2.4	479	37.9	10.784	6.232	673.7	81.00
	3.2	500	31.3	9.622	8.018	787.6	84.36
BNSB02	0.8	457	45.6	17.559	6.489	396.8	77.22
	1.6	464	28.5	9.654	7.696	880.2	85.77
	2.4	489	22.6	10.677	9.243	1002.7	88.67
	3.2	501	17.6	11.398	9.475	1184.9	91.21
BNSB03	0.8	467	60.2	15.481	5.963	336.9	69.92
	1.6	481	46.5	17.545	6.291	392.5	76.77
	2.4	491	34.9	12.863	7.691	606.8	82.58
	3.2	481	29.7	12.281	8.440	707.2	85.17

The percentage inhibition efficiency was calculated using eqn (8):8
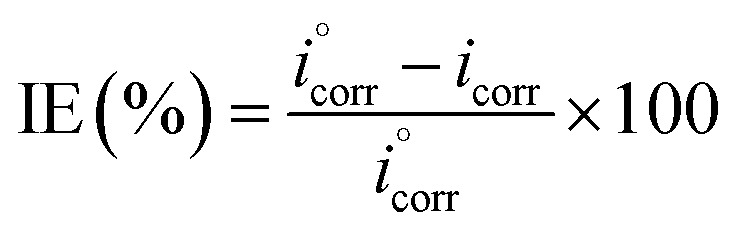
where 
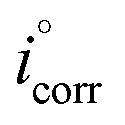
 and *i*_corr_ are the corrosion current densities for blank and in the presence of inhibitors, respectively. The corrosion current density for mild steel without any inhibitor was 0.2 mA cm^−2^, which was higher than values recorded in the presence of inhibitors. The decrease in *i*_corr_ value for all three inhibitors indicated that mild steel corrosion was inhibited with an increase in the concentration of the synthesized Schiff bases. Although both cathodic and anodic slopes were displaced in the presence of inhibitors, the cathodic slope showed slightly less displacement compared to the anodic slope. This showed that the kinetics of the dissolution of metal and the evolution of hydrogen changed with the addition of BNSB01, BNSB02, and BNSB03, whereas the reduction of iron was more affected. The values of *b*_c_ and *b*_a_ did not show any regular pattern, which indicated that apart from adsorption, a number of other corrosion-inhibiting mechanisms were taking place.^39^ The variation in the values of *b*_a_ and *b*_c_ was also caused by the interference of other species present during inhibitor adsorption.^40^ Another reason for this variation might be the lack of a detectable Tafel region. With the addition of an inhibitor, an anodic shift in the equilibrium potential value was observed. Corrosion inhibitors are classified as anodic or cathodic based on whether the shift in *E*_corr_ is more than ±85 mV relative to the *E*_corr_ value of the metal in the uninhibited solution.^41^ In this study, the shift in the value of *E*_corr_ was less than ±85 mV, and hence the synthesized Schiff bases were classified as mixed-type inhibitors. In the uninhibited solution, the value of linear polarisation resistance was 302 Ω cm^2^, which was lower than the values measured in the presence of inhibitors at all concentrations. On increasing the concentration of inhibitors, the value of LPR increased, and the maximum value of 1184.9 Ω cm^2^ was observed for BNSB02 when concentration was 3.2 mM.

### 3.4 Electrochemical impedance spectroscopy

**Fig. 5:**
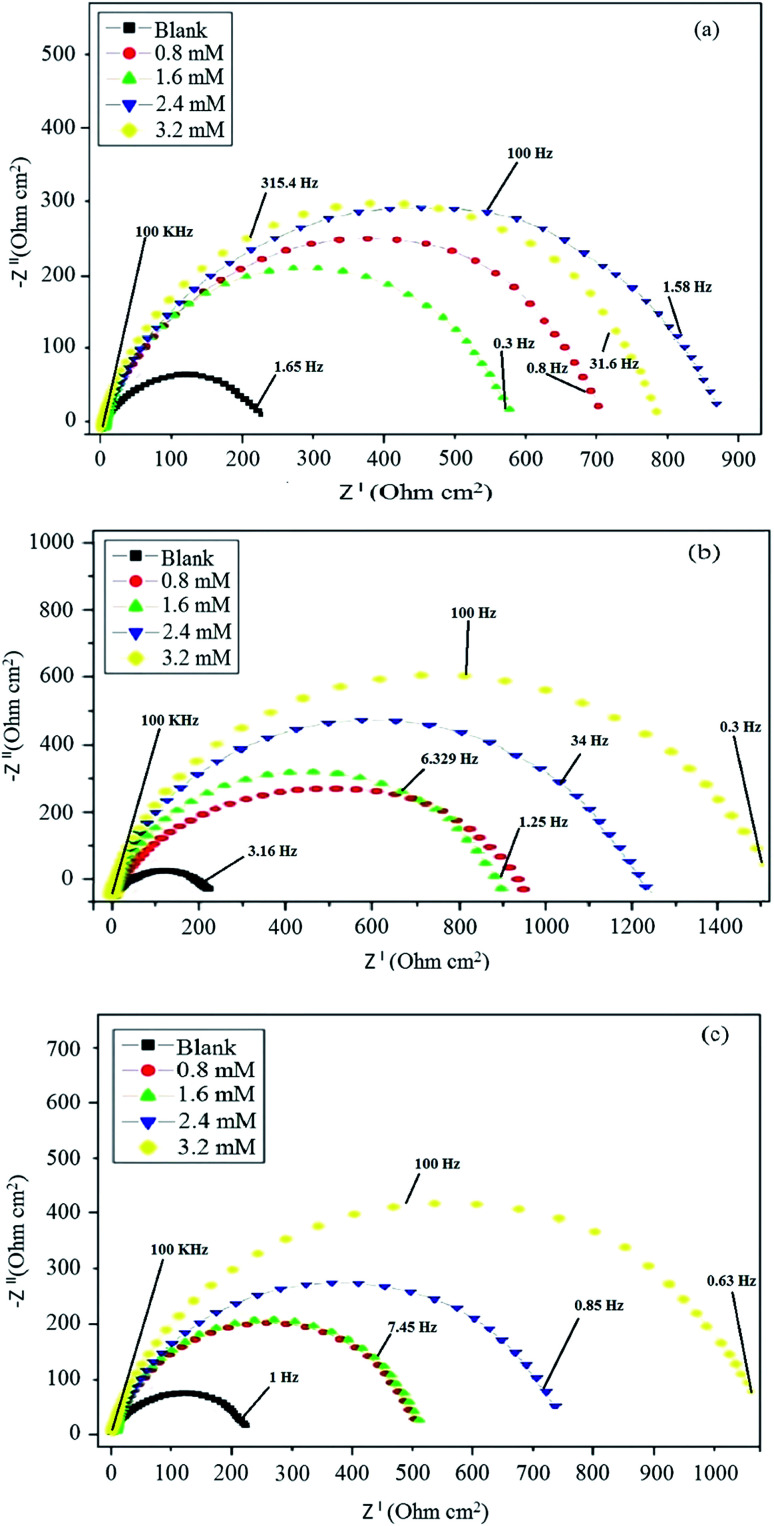
Nyquist plots in the absence and presence of different concentrations of (a) BNSB01, (b) BNSB02 and (c) BNSB03.

The corrosion behavior of mild steel in 0.5 M hydrochloric acid solutions containing different concentrations of Schiff base inhibitors was studied by electrochemical impedance spectroscopy (EIS). The Nyquist plots of mild steel in the acidic solution in the absence and presence of inhibitors consisted of capacitive loops, as presented in Fig. 5. It was observed that the capacitive loops were imperfect semicircles, which might be due to the presence of impurities, inhomogeneity on the surface of the metal in terms of roughness, frequency of dispersion, distribution of surface-active sites and grain boundaries. Therefore, to get a more accurate fit, a constant phase element (CPE) was introduced into the circuit.^42,43^ The presence of a single semicircle during the dissolution of metal corresponds to a single charge transfer reaction. For the description of a frequency independent phase shift between an applied ac potential and its current response, a constant phase element (CPE) is used which is defined in impedance representation as in the following equation eqn (9).

**Fig. 6:**
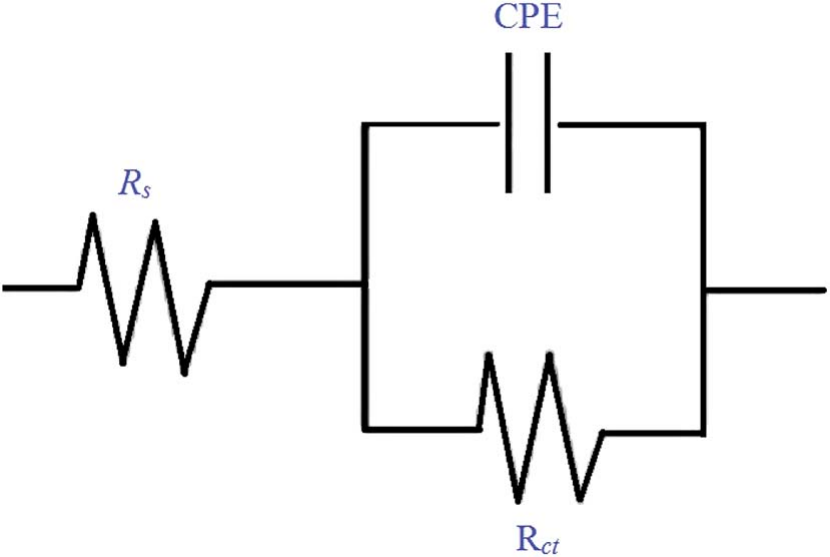
Equivalent circuit model used to fit the EIS data.

9 *Z*_CPE_ = *Y*_o_^−1^(i*ω*)^−1^

where *Y*_o_ is the CPE constant, *ω* is the angular frequency (in rad s^−1^), i^2^ = −1 is the imaginary number and *n* has the meaning of phase shift.^25^ The value of *n*, which is the measure of surface inhomogeneity, was found to increase from 0.7 to 0.95 and showed a deviation from the ideal behavior, for which *n* is equal to 1. Based on the equivalent circuit shown in Fig. 6, the Nyquist plots were explained to consist of the charge transfer resistance (*R*_ct_) connected in parallel to the constant phase element (CPE) and both connected in series with the solution resistance (*R*_s_). Table 5 shows the values of all the studied parameters.

**Table 5:** Impedance parameters for the corrosion of mild steel in 0.5 M HCl in the absence and presence of different concentrations of BNSB01, BNSB02 and BNSB03 at 303 K

Inhibitor	Concentration (mM)	*R*_ct_ (Ω cm^2^)	*Y*_o_ (μΩ^−1^ s^*n*^)	*R*_s_ (Ω cm^2^)	*n*	IE (%)
	Blank	205	275.6	2.471	0.7631	—
BNSB01	0.8	545.6	78.29	6.606	0.8457	62.42
	1.6	614	102.4	1.151	0.8339	66.61
	2.4	726.1	65.66	1.245	0.8637	71.76
	3.2	754.6	95.37	2.856	0.8164	72.83
BNSB02	0.8	780.3	75.53	2.411	0.8036	73.72
	1.6	837.2	44.41	3.645	0.8705	75.51
	2.4	1106	20.15	1.205	0.9207	81.46
	3.2	1372	19.32	1.194	0.9211	85.05
BNSB03	0.8	497.2	94.57	7.513	0.8513	58.76
	1.6	501.7	84.87	7.522	0.8633	59.13
	2.4	698.9	76.19	4.645	0.8430	70.66
	3.2	1030	69.64	3.120	0.8533	80.09

Charge transfer resistance and inhibition efficiency are directly related to each other. The value of charge transfer resistance *R*_ct_ was obtained by the difference between the real impedance at the lower and higher frequencies. Charge transfer resistance increased with an increase in adsorption on the surface of the metal. This was due to the increase in the concentration of the inhibitors, which also corresponded to the increase in the diameter of the semicircle. By using eqn (10), the percentage inhibition efficiency was calculated as:10
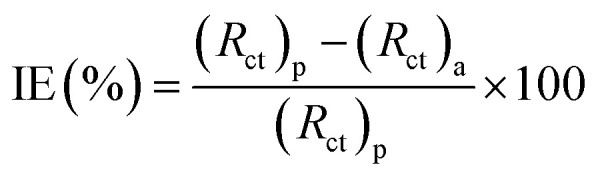
where (*R*_ct_)_a_ and (*R*_ct_)_p_ are the charge transfer resistances of the blank and in the presence of inhibitors, respectively. The value of *R*_ct_ increased to 1372 Ω cm^2^ for BNSB02 at 3.2 mM and 205 Ω cm^2^ for blank. The values of *Y*_o_ for all the three synthesized Schiff base inhibitors at all concentrations were less compared to the CPE constant *Y*_o_ of the blank, which was 275.60 μΩ^−1^ s^*n*^. The decrease in the value of *Y*_o_ on the addition of inhibitors might be due to the desorption of water from the surface of mild steel. This would be followed by adsorption of inhibitor and result in an increase in the double layer thickness due to the Schiff bases on the surface of metal or a decrease in the local dielectric constant. The large values of *n* for all the Schiff bases represent reduced inhomogeneity owing to the formation of a protective film.

**Fig. 7:**
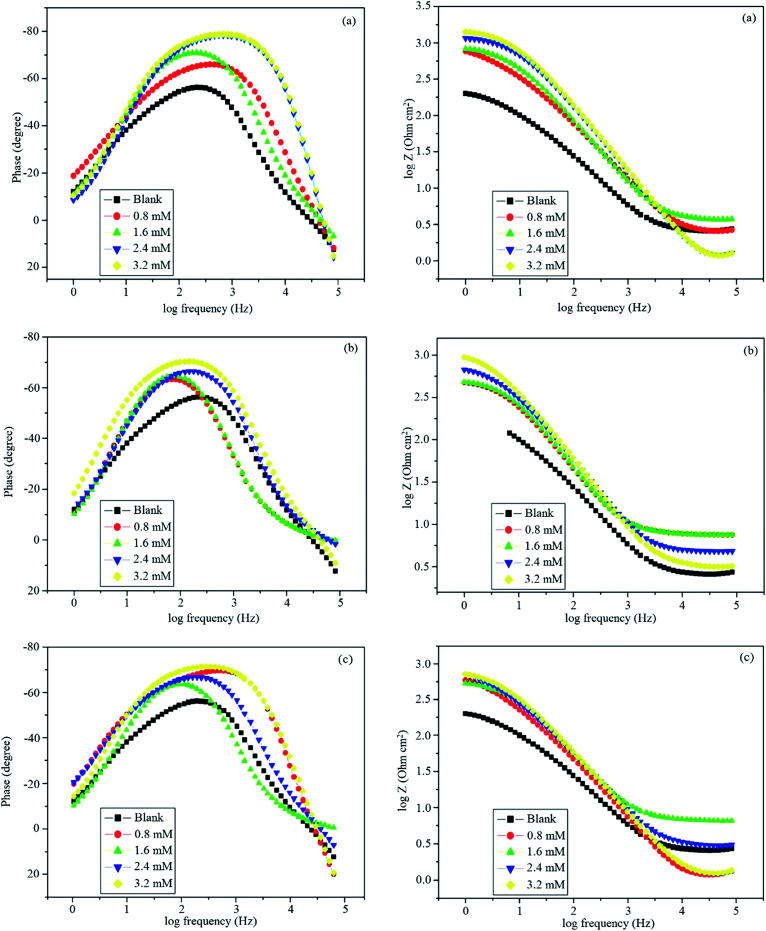
Bode plots in the absence and presence of different concentrations of (a) BNSB01, (b) BNSB02 and (c) BNSB03.

After immersing mild steel in 0.5 M HCl, Bode plots were recorded for blank and in the presence of inhibitors and are shown in Fig. 7. At higher concentrations of inhibitors, a phase angle shift was observed, which might be due to the protective layer formed on the surface of mild steel that alters the interfacial structure of the electrode.^44^ The phase shift was more at higher concentrations because a greater number of Schiff base molecules occupied the large surface area. With increasing concentration, an increase in the value of impedance increased the tendency of current passing through the capacitor.

## 4. Mechanism of inhibition

**Fig. 8:**
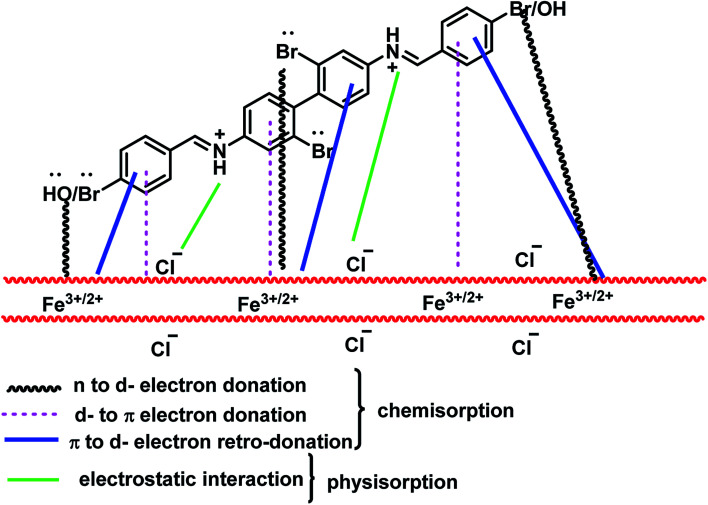
Proposed model of adsorption of the synthesized Schiff base inhibitors on mild steel in 0.5 M HCl.

The inhibition efficiency of BNSB01, BNSB02 and BNSB03 on mild steel in 0.5 M HCl can be explained on the basis of molecular size; the modes of interaction of the Schiff bases with the metal surface include the nature of bonds in the metal along with their capability to produce complexes (chemisorption) and the number of adsorption sites (physisorption). Since all the three Schiff bases have at least two nitrogen atoms, two bromine atoms, and π-electrons, all of these act as adsorption centers. Therefore, inhibitors make coordinate covalent bonds using these electrons and chemisorb onto the surface of mild steel. It is obvious that the protonation of a nitrogen atom is very easy, and it can be physically adsorbed *via* a chloride ion (Cl^−^). The large inhibition efficiency of BNSB02 and BNSB03 might be due to the presence of the additional electron-releasing bromine (Br–) and hydroxyl (–OH) groups, respectively. The experimental and quantum chemical calculation studies suggested that the inhibition efficiency of the studied Schiff bases followed the order: BNSB02 > BNSB03 > BNSB01. The schematic representation of the different modes of adsorption of the most strongly adsorbed Schiff base BNSB02 on the metal/acid interface is shown in Fig. 8. From the above study, it is obvious that the lone pair electrons of the bromine atoms are donated to the d-orbital of Fe, and therefore, the highest inhibition efficiency is shown by BNSB02 due to the presence of four bromine atoms. Such donation might cause the build-up of excessive negative charge on the mild steel surface, which facilitates the transfer of electrons from the d-orbitals of the metal to the π-antibonding molecular orbitals of the Schiff bases by retro-donation. These donations and retro-donations support each other through synergy.^45^ The lowest inhibition efficiency was exhibited by BNSB01 compared to the other two inhibitors because BNSB02 and BNSB03 possess a bromo substitute on one aryl group and bromo or hydroxy substituents on other the aryl group, while BNSB01 has no electron releasing groups (Br, OH) on its terminal aryl groups.

## 5. Morphological investigation

**Fig. 9:**
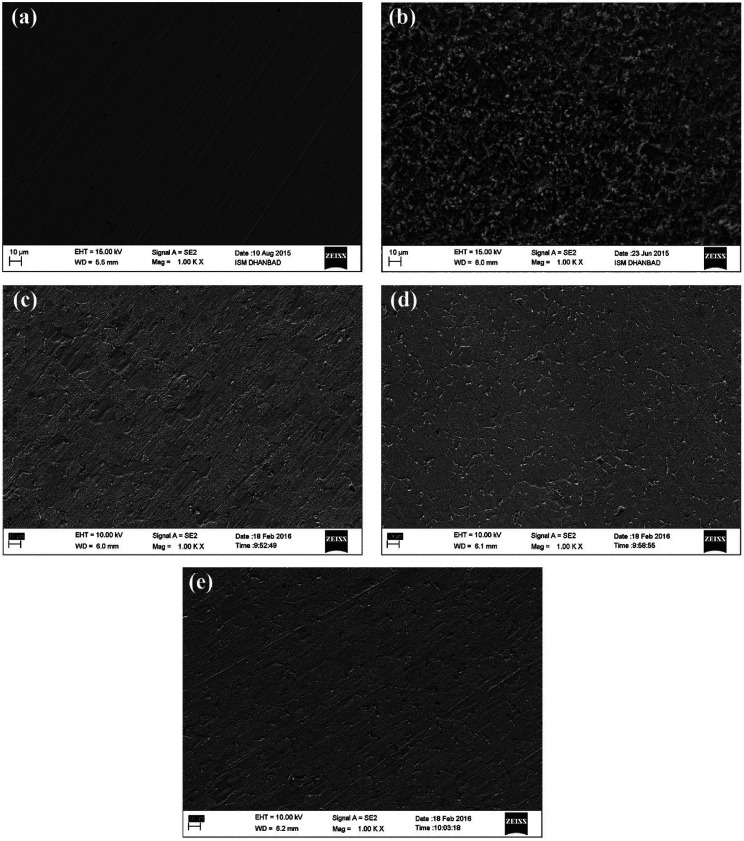
SEM images of mild Steel after 4 h immersion in 0.5 M HCl at 30 °C: (a) before immersion (polished), (b) with 0.5 M HCl without inhibitor, (c) with 3.2 mM BNSB01, (d) with 3.2 mM BNSB02 and (e) with 3.2 mM BNSB03.

To confirm the adsorption of the three inhibitors on the mild steel surface in the presence and absence of inhibitors in 0.5 M HCl, field emission-scanning electron microscopy (FE-SEM) experiments were carried out. Fig. 9 demonstrates the Fe-SEM images of the mild steel surface without and with the acidic solution as well as in the presence of BNSB01, BNSB02 and BNSB03 at the optimum concentration of 3.2 mM at 30 °C. Fig. 9a is the surface of mild steel before immersion in 0.5 M HCl, while Fig. 9b is the image of the surface after 4 h immersion in the acidic solution. It was clear from the FE-SEM images that the mild steel surface was highly corroded because of the aggressive acidic condition. There were several swollen structures (Fig. 9b), which showed the direct attack of the aggressive acidic ions. However, the images in Fig. 9c–e showed a smooth surface in the presence of inhibitors with no significant change except emery traces, which indicated the high degree of corrosion protection to the mild steel surface offered by the synthesized inhibitors.

## 6. Conclusion

In summary, we demonstrated the synthesis of three bis-Schiff bases BNSB01, BNSB02 and BNSB03 and examined their potential to act as mixed-type inhibitors. All the tested compounds showed excellent inhibition efficiency for mild steel in 0.5 M HCl. The analysis of weight loss showed that the inhibition efficiency increased with an increase in the concentration of inhibitors and decreased with an increase in temperature. The study of adsorption isotherms and thermodynamic parameters revealed physisorption, and the isotherm was the Langmuir type. Polarisation studies revealed that the inhibitors affected both cathodic, as well as anodic reactions. Electrochemical impedance studies concluded that the large values of charge transfer resistance for the inhibitor solutions resulted in their higher inhibition efficiency. Further, the SEM images, which illustrated the formation of a protective layer on the surface of mild steel, supported the corrosion inhibition activity of these bis-Schiff bases.

## Conflicts of interest

There are no conflicts to declare.

## Supplementary Material

RA-010-C9RA06443E-s001
