# Supplementary material for: Bis-Schiff bases of 2,2′-dibromobenzidine as efficient corrosion inhibitors for mild steel in acidic medium
Source: RSC Adv. 2020 Jan 28;10(8):4499–511. doi: 10.1039/c9ra06443e (PMC9049013; doi:10.1039/c9ra06443e)
Supplement: RA-010-C9RA06443E-s001 [file RA-010-C9RA06443E-s001.pdf]

Electronic Supplementary Material (ESI) for RSC Advances.
This journal is © The Royal Society of Chemistry 2020

SUPPLEMENTARY DATA

Bis-Schiff bases of 2,2'-Dibromobenzidine as an efficient corrosion inhibitor for mild steel in acidic medium

Ifzan Arshad*[a,b], Aamer Saeed*[a] , Pervaiz Ali Channar[a], Syeda Aaliya Shehzadi[a,c], Muhammad Naeem Ahmed[d] and Muhammad Siddiq[a]

[a] *Department of Chemistry, Quaid-I-Azam University, Islamabad 45320, Pakistan*

[b] *CAS Key Laboratory of Materials for Energy Conversion, Department of Materials Science and Engineering, University of Science and Technology of China (USTC), JinZhai Road, Hefei, Anhui Province 230026, P. R. China*

[c] *Sulaiman Bin Abdullah Aba Al-Khail-Centre for Interdisciplinary Research in Basic Sciences (SA-CIRBS) International Islamic University, Islamabad, Pakistan*

[d] *Department of Chemistry, The University of Azad Jammu and Kashmir Muzaffarabad, 13100, Pakistan*

Corresponding author E-mail: mifzan@gmail.com (Ifzan Arshad), aamersaeed@yahoo.com (Dr. Aamer Saeed)

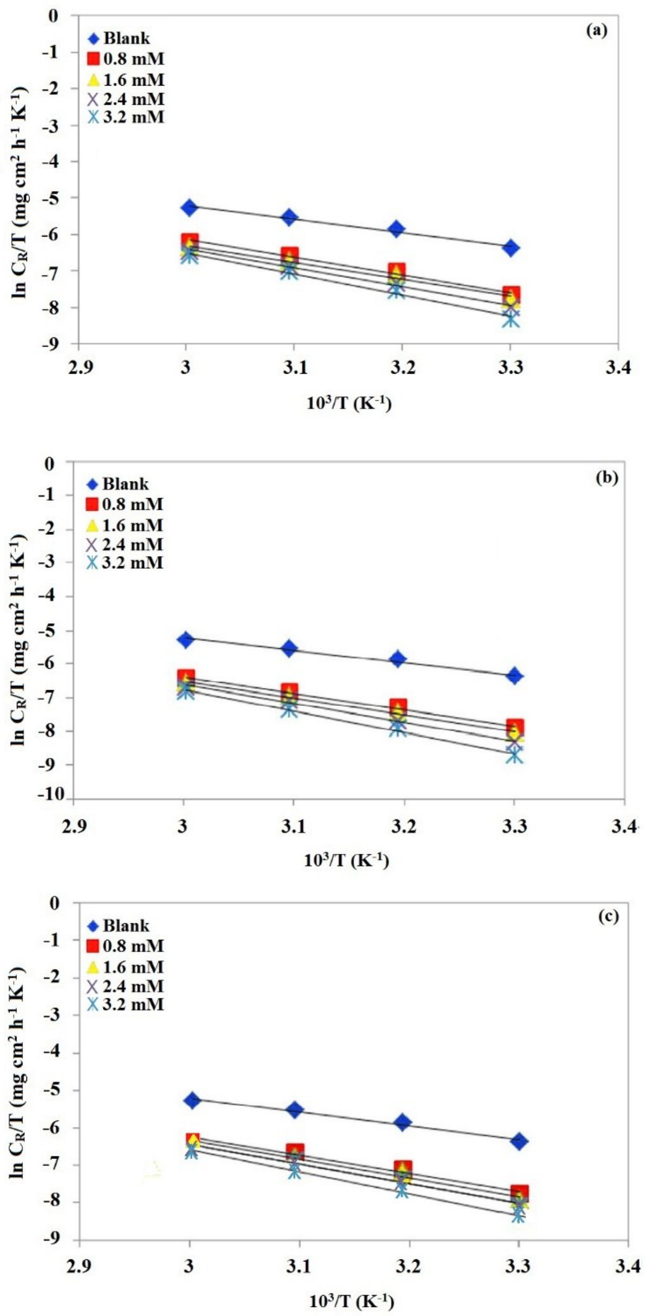

Fig. S1. Alternative Arrhenius plots for mild steel in 0.5 M HCl in the absence and presence of different concentrations of (a) BNSB01 (b) BNSB02 and (c) BNSB03.

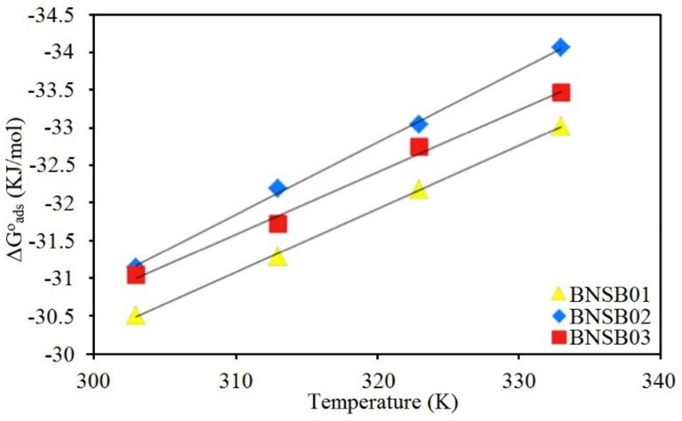

Fig. S2. Plot of ΔG_{ads} vs. T for BNSB01, BNSB02 and BNSB03.

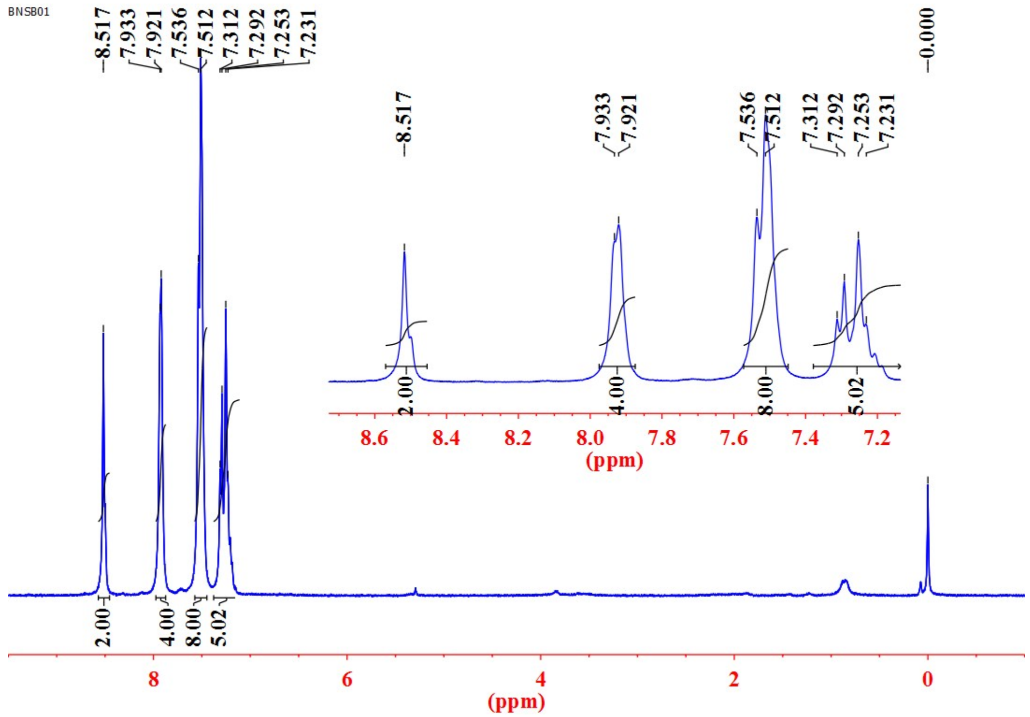

Fig.S3 ^{1}H NMR of **BNSB01**

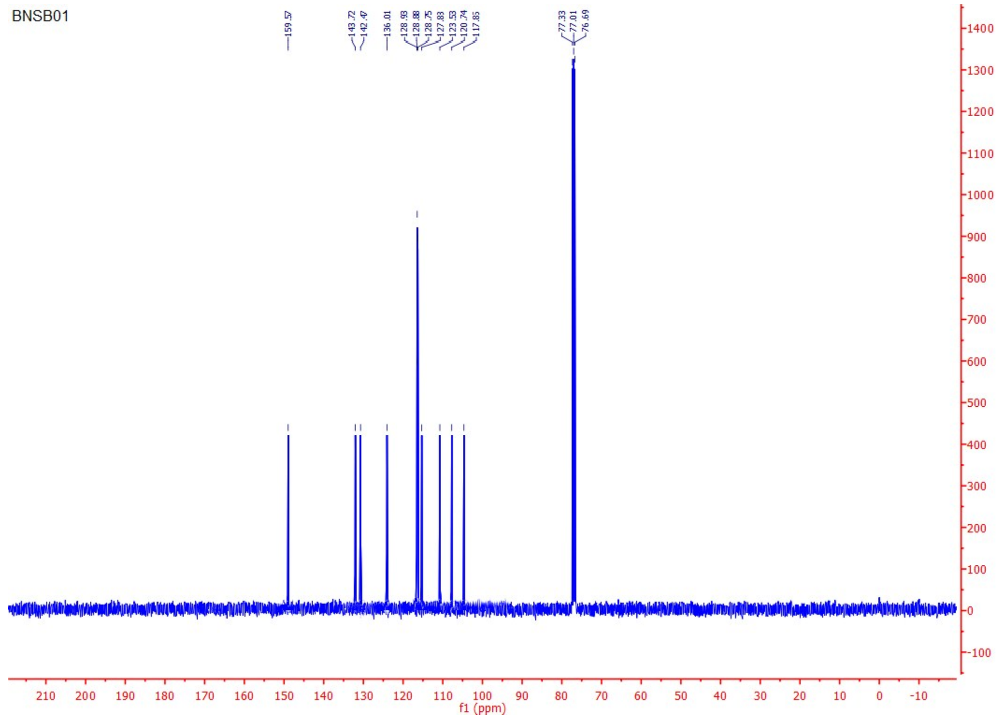

Fig.S4 ^{13}C NMR of **BNSB01**

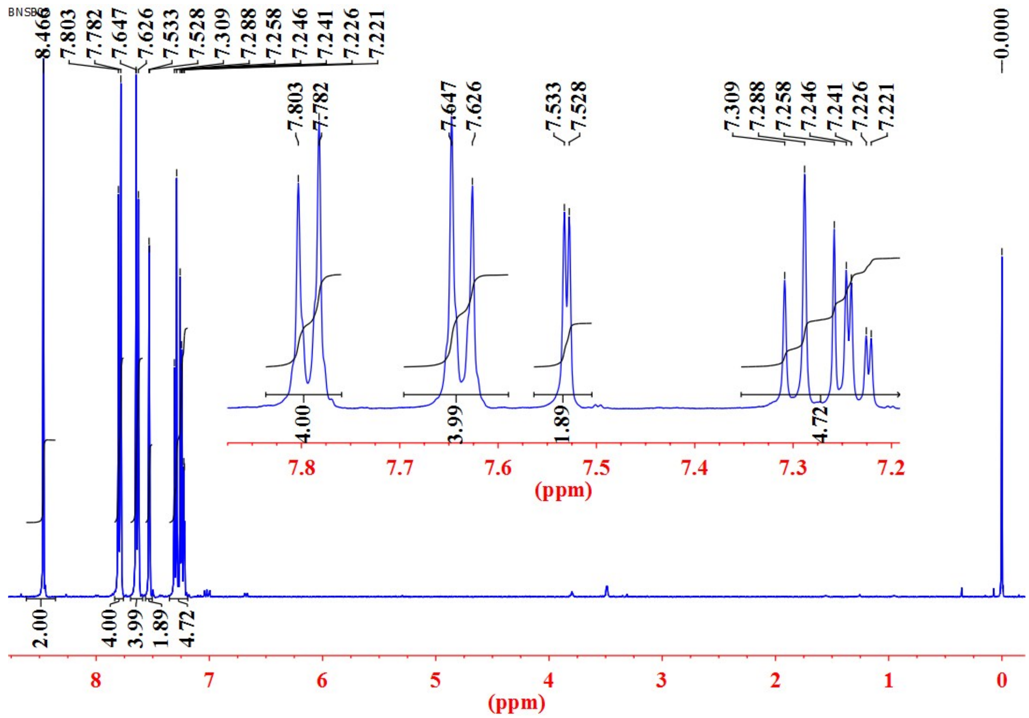

Fig.S5 ^{1}H NMR of **BNSB02**

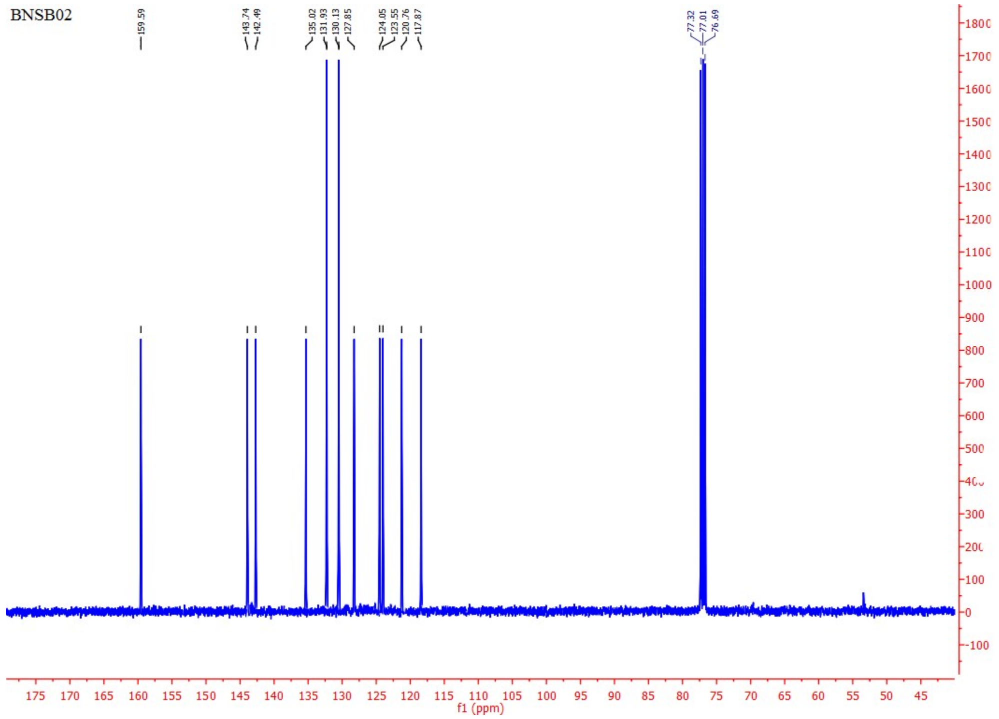

Fig.S6 ^{13}C NMR of **BNSB02**

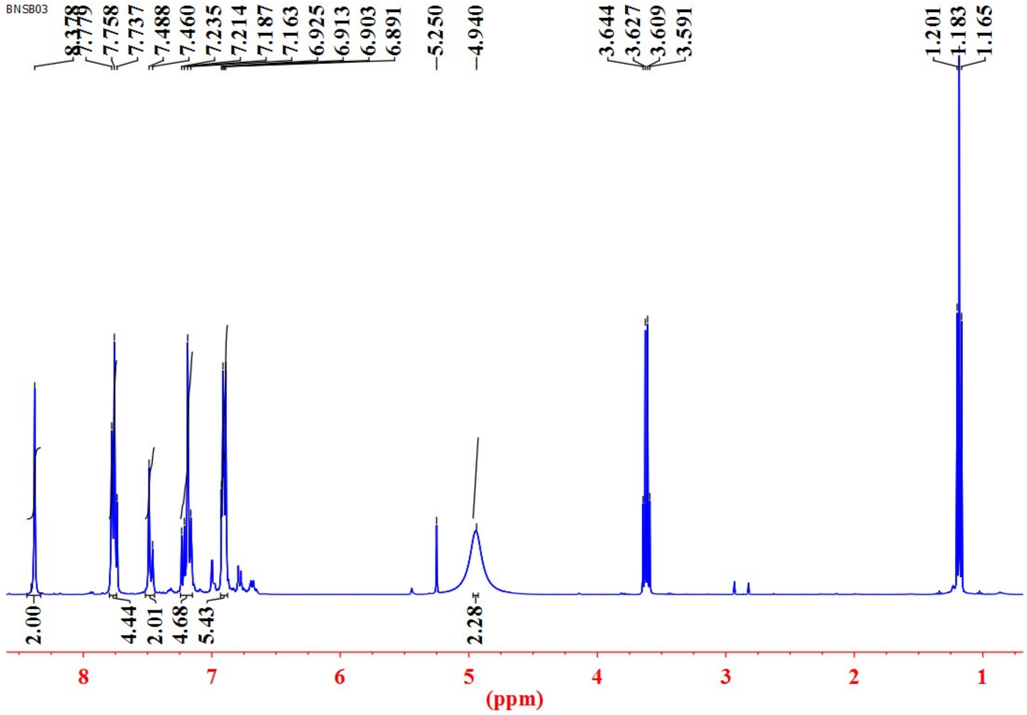

Fig.S7 ^{1}H NMR of **BNSB03**

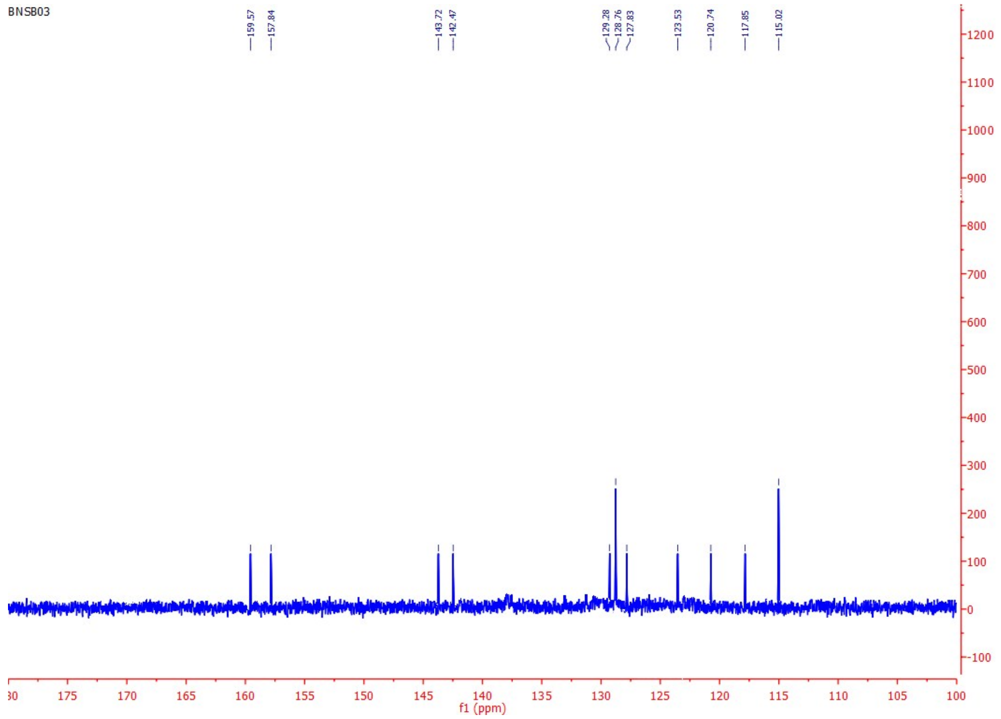

Fig.S8 ^{13}C NMR of **BNSB03**
